# Bie-Jia-Ruan-Mai-Tang, a Chinese Medicine Formula, Inhibits Retinal Neovascularization in Diabetic Mice Through Inducing the Apoptosis of Retinal Vascular Endothelial Cells

**DOI:** 10.3389/fcvm.2022.959298

**Published:** 2022-07-12

**Authors:** Qiu-Ping Liu, Yu-Ying Chen, Yuan-Yuan Yu, Pei An, Yi-Zhuo Xing, Hong-Xuan Yang, Yin-Jian Zhang, Khalid Rahman, Lei Zhang, Xin Luan, Hong Zhang

**Affiliations:** ^1^Shanghai Frontiers Science Center of TCM Chemical Biology, Institute of Interdisciplinary Integrative Medicine Research, Shanghai University of Traditional Chinese Medicine, Shanghai, China; ^2^Ophthalmology Department of Longhua Hospital, Shanghai University of Traditional Chinese Medicine, Shanghai, China; ^3^School of Pharmacy and Biomolecular Sciences, Faculty of Science, Liverpool John Moores University, Liverpool, United Kingdom; ^4^Department of Vascular Surgery, Yueyang Hospital of Integrated Traditional Chinese and Western Medicine, Shanghai University of Traditional Chinese Medicine, Shanghai, China

**Keywords:** Chinese medicine formula, neovascularization, mitochondrial pathway, apoptosis, diabetic retinopathy

## Abstract

Proliferative diabetic retinopathy (PDR) is one of the main complications of diabetes, mainly caused by the aberrant proliferation of retinal vascular endothelial cells and the formation of new blood vessels. Traditional Chinese medicines possess great potential in the prevention and treatment of PDR. Bie-Jia-Ruan-Mai-Tang (BJ), a Chinese medicine formula, has a good therapeutic effect on PDR clinically; however, the mechanism of action involved remains unclear. Therefore, we investigated the effect of BJ on PDR through *in vitro* and *in vivo* experiments. A diabetic mouse model with PDR was established by feeding a high-fat–high-glucose diet combined with an intraperitoneal injection of streptozotocin (STZ), while high-glucose-exposed human retinal capillary endothelial cells (HRCECs) were employed to mimic PDR *in vitro*. The *in vivo* experiments indicated that BJ inhibited the formation of acellular capillaries, decreased the expression of VEGF, and increased the level of ZO-1 in diabetic mice retina. *In vitro* experiments showed that high glucose significantly promoted cell viability and proliferation. However, BJ inhibited cell proliferation by cycle arrest in the S phase, thus leading to apoptosis; it also increased the production of ROS, decreased the mitochondrial membrane potential, reduced the ATP production, and also reduced the expressions of p-PI3K, p-AKT, and Bcl-xL, but increased the expressions of Bax and p-NF-κB. These results suggest that BJ induces the apoptosis of HRCECs exposed to high glucose through activating the mitochondrial death pathway by decreasing the PI3K/AKT signaling and increasing the NF-κB signaling to inhibit the formation of acellular capillaries in the retina, thus impeding the development of PDR.

## Introduction

Diabetes mellitus is a common metabolic disease characterized by chronic hyperglycemia, which can cause multiple organ damage (1). The development of hyperglycemia into diabetic complications is a complex process involving a series of mechanisms, and currently, no specific treatment is available (2). It was reported that there were 382 million diabetic patients worldwide in 2013, and moreover, this number is expected to increase to 592 million by 2035 (3). Diabetes can elicit a variety of complications, such as cardiovascular, kidney, and eye diseases, resulting in great inconvenience, economic pressure, and a sharp decline in the quality of life for the patients (4). A total of 14.8% of diabetic patients have eye complications, among which diabetic retinopathy (DR) is the most common and can lead to blindness (5). In total, 35% of patients eventually develop some form of retinopathy, suggesting that DR has the potential to be the leading cause of visual impairment and blindness worldwide (6). Therefore, it is essential to develop drugs for the prevention and treatment of DR. At present, panretinal photocoagulation (PRP) and anti-VEGF chemical drugs are the main treatment. Although these therapies indeed play a positive role in some aspects, however, the associated side effects cannot be ignored. For example, PRP can cause peripheral vision loss, night blindness, choroidal effusion, and macular edema, while the persistent effect is limited for the anti-VEGF chemical drugs. The current therapies still need to be improved for efficacy, safety, and persistence (7).

Owing to the characteristics of multiple targets, active components, and good safety, Chinese medicines have received increasing attention from clinicians and researchers worldwide (8). Numerous investigations have shown that Chinese medicines have great potential in the prevention and treatment of diabetes (9). Bie-Jia-Ruan-Mai-Tang (BJ) is an empirical prescription based on traditional Chinese medicine theory and long-term clinical practice, which is well summarized by Professor Jiu-Yi Xi, a famous expert in the treatment of peripheral vascular disease from the Yueyang Hospital of Integrated Traditional Chinese and Western Medicine Affiliated to the Shanghai University of Traditional Chinese Medicine. BJ is composed of *Trionyx sinensis* Wiegmann, the rhizome of *Acorus tatarinowii*, the whole plant of *Sedum sarmentosum*, and the root of *Paeonia lactiflora*, and has been clinically used for decades for the treatment of diabetic vascular complications in the Yueyang Hospital of Integrated Traditional Chinese and Western Medicine and Longhua Hospital affiliated to the Shanghai University of Traditional Chinese Medicine because of its good efficacies in softening hardness, relieving spasm, clearing heat, detoxifying, invigorating qi, and promoting blood circulation (10, 11).

Endothelial dysfunction reflects an imbalance of endothelial cell-derived active substances, which can elicit injury, activation, and inflammation of endothelial cells. In addition, the dysfunction of endothelial cells is one of the main reasons for DR (12, 13). Although BJ has been used clinically for decades, the related mechanism of action still remains unclear. Accordingly, the present investigation aimed to verify the effects of BJ on diabetic mice with retinopathy and HRCECs exposed to high glucose, and to explore the possible mechanism of action involved.

## Materials and Methods

### Preparation of Bie-Jia-Ruan-Mai-Tang Extract

The raw herbal materials comprising (BJ; the composition is shown in Table 1) were provided by the Ophthalmology Department of Longhua Hospital affiliated to the Shanghai University of Traditional Chinese Medicine, which were identified by Professor Hai-Liang Xin, a pharmacognosist in the Naval Military Medical University (Shanghai, China). The voucher specimen was deposited in the System Pharmacology Research Center, Institute of Interdisciplinary Integrative Medicine Research, Shanghai University of Traditional Chinese Medicine (SP2020016). These herbal materials were extracted twice for 1.5 h by refluxing with eight times the amount of 75% ethanol. The extracted solution was concentrated in a rotating evaporator after filtering with a four-layer gauze, and then pre-freeze at −50°C for 5 h of vacuum dry in the material tray of a freeze-dryer. The temperature was finally fixed at −40°C for 72 h to make a lyophilized powder. Finally, the extract with a yield of 18% was obtained and stored in a refrigerator at −80°C until used.

**TABLE 1:** The raw herbal material composition of Bie-Jia-Ruan-Mai-Tang.

Name of herbal material	Proportion
Shell of *Trionyx sinensis* Wiegmann	10
Whole plant of *Sedum sarmentosum* Bunge	6
Rhizome of *Acorus tatarinowii* Schott	6
Root of *Paeonia lactiflora* Pall	9
Root of *Astragalus mongholicus* Bunge	9
Root of *Dipsacus eroides* C. Y. Cheng et T. M.	6
Root and rhizome of *Glycyrrhiza uralensis* Fisch.	3
Flower bud and inflorescence of *Buddleja officinalis* Maxim.	6

### Reagent

The cell-counting kit-8 (CCK-8) was purchased from the Meilun Biotechnology Co., Ltd. (Dalian, China). The ATP assay kit, the mitochondrial membrane potential kit, and the ROS assay kit were obtained from the Beyotime Biotechnology (Shanghai, China). The kits for the detection of cell apoptosis and cycle were provided by the KeyGen Biotechnology Co., Ltd. (Nanjing, China). Primary antibodies of p-NF-κB, NF-κB, p-PI3K, PI3K, p-AKT, AKT, BCL-XL, BAX, and GAPDH for Western blot were bought from the Cell Signaling Technology (Danvers, United States).

### Animal Experiment

Male C57BL/6J mice (8 weeks of age) weighing 22–24 g were provided by the Shanghai Model Organisms Center, Inc. The animals were housed in a temperature-controlled room (23 ± 2°C) under a 12:12 h light–dark cycle of artificial light, with free access to food and water. The establishment of the mice model with DR was conferred with previous reports with slight alterations (14). Briefly, after 1 week of adaptive feeding, the animals were fed a high-fat–high-glucose diet (comprising 58.8% high-nutrition base feed, 20% glucose, 20% lard, 1% total cholesterol, and 0.2% sodium cholate, and purchased from the Nanjing Shengmin Scientific Research Animal Farm) for 17 weeks. Another eight mice given an ordinary diet were used as normal control. After 4 weeks, mice fed a high-fat–high-glucose diet were intraperitoneally injected with 50 mg/kg STZ (Sigma-Aldrich, St. Louis, MO, United States) for 5 consecutive days, while those in the normal control were injected with the same volume of normal saline. Four weeks after injection, the mice with blood glucose concentration above 16.7 mmol/L were considered to be type 2 diabetics [Shen et al. (15)]. The diabetic mice were divided into five different groups (*n* = 8 each group), including control group (Diabetic group), calcium dobesilate group (CAD, 0.25 g/kg.day^–1^), low-dose group (LDG, 1.6 g/kg.day^–1^ BJ) group, medium-dose group (MDG, 3.2 g/kg.day^–1^ BJ), and high-dose group (HDG, 6.4 g/kg.day^–1^ BJ). Eight weeks after the administration of the above compounds, the mice were sacrificed by ether anesthesia, and the eyeballs were removed and stained with hematoxylin and eosin (H and E) and PAS (Periodic Acid-Schiff), and immunohistochemistry analyses were performed.

All animal experiments were approved by the Experimental Animal Ethics Committee of the Shanghai University of Traditional Chinese Medicine and performed in compliance with the University’s Guidelines for the Care and Use of Laboratory Animals. The ethical number PZSHUTCM200814008 was adopted on 14 August 2020.

### Cell Culture

Human retinal capillary endothelial cells (HRCECs) were purchased from the Cell Bank of Shanghai Academy of Chinese Sciences (Shanghai, China) and maintained in DMEM (HyClone, United States) supplemented with 10% FBS (GIBCO, United States) in an incubator of 5% CO_2_ at 37°C. The HRCECs were then divided into two groups, *viz*. normal group of cells cultured in 5.5 mM glucose medium (NG) and a high-glucose group of cells cultured in 35 mM glucose medium (HG). In subsequent experiments, high-glucose-cultured cells were treated with BJ extract. A total of 0.1 g BJ extract was dissolved in 1 mL dimethyl sulfoxide, and the solution was filtered with a 0.22 μm sterile microporous membrane and diluted to the corresponding concentrations with the medium.

### Cell-Counting Kit-8 Assay

Human retinal capillary endothelial cells cultured in NG and HG were, respectively, seeded into 96-well plates (5 × 10^3^ cells/well), incubated at 37°C and 5% CO_2_ for 24 h. The supernatant was discarded, and then the corresponding medium and different concentrations of BJ (10, 25, 50, 100, and 150 μg/mL) were added. After treatment with BJ for 24 h or 48 h, the supernatant was discarded, 100 μL of 10% CCK8 was added and incubated at 37°C for 30 min, and the absorbance was measured at 450 nm using a microplate reader. Cell viability was calculated as follows: Cell viability rate (%) = (absorbance of the experimental group–absorbance of the blank group)/(absorbance of the control group–absorbance of the blank group) × 100%.

### Cell Colony Formation

Human retinal capillary endothelial cells cultured in NG and HG were, respectively, seeded into the 6-well plates (600 cells/well) and incubated at 37°C and 5% CO_2_ for 24 h. The supernatant was discarded and the different concentrations of BJ (50, 100 μg/mL) were added to six-well plates for 8 consecutive days of incubation. Following this, HRCECs were fixed with 4% paraformaldehyde for 30 min and stained with crystal violet for 15 min, HRCECs cultured in NG were used as control.

Clone formation rate (%) = (Number of clones/number of inoculated cells) × 100%.

### Cell Cycle Analysis

Human retinal capillary endothelial cells cultured in NG and HG were, respectively, harvested and seeded into six-well plates (5 × 10^5^ cells/well) and incubated at 37°C for 24 h in the presence of 5% CO_2_. After discarding the supernatant, different concentrations of BJ (50 and 100 μg/mL) or corresponding medium were added for another 24 h of incubation. One milliliter of precooled 70% ethanol was added to each well, and the cells were fixed at 4°C for 2 h. The cells were then collected, mixed with 0.5 mL PI, and the samples were incubated in the dark for 30 min, and the Beckman flow cytometer was used for detection.

### Apoptosis Detection

Human retinal capillary endothelial cells cultured in NG and HG were, respectively, harvested and seeded into six-well plates (5 × 10^5^ cells/well) and incubated at 37°C, for 24 h, in the presence of 5% CO_2_. The different concentrations of BJ (50 and 100 μg/mL) were added for another 24 h of incubation. The cells were digested and centrifuged at 2,000 rpm for 10 min, 100 μL 1 × FITC binding solution was added to each well, mixed, and then 5 μL FITC dye was added for 10 min of incubation at room temperature in the dark. Finally, 5 μL PI dye was added for 5 min and the samples were incubated in the dark. HRCECs cultured in NG were used as control and the Beckman flow cytometer was used for detection.

### JC-1 Mitochondrial Membrane Potential

Human retinal capillary endothelial cells cultured, respectively, in NG and HG were harvested and seeded into 24-well plates (5 × 10^4^ cells/well) and incubated at 37°C, for 24 h, in the presence of 5% CO_2_. After removing the supernatant, the corresponding medium or different concentrations of BJ (50 and 100 μg/mL) were added for another 24 h of incubation. The transformation from red fluorescence to green fluorescence was used as one of the early detection indicators for cell apoptosis, and the fluorescence quantification was carried out using the Image J software.

### Adenosine Triphosphate Detection

Adenosine triphosphate concentration in HRCECs was detected by the use of an ATP Assay Kit. Briefly, HRCECs cultured in NG and HG were, respectively, harvested, seeded into six-well plates (5 × 10^5^ cells/well), and incubated at 37°C for 24 h in the presence of 5% CO_2_. The corresponding medium or different concentrations of BJ were then added for 24 h of incubation. Cell lysis solution was added and the samples were centrifugated for 10 min at 12,000 × *g* at 4°C. A total of 20 μL sample or standard solution was added to 100 μL of ATP detection solution, mixed, and then luminescence was measured with a multifunctional enzyme plate analyzer.

### Cell Nuclear Staining

Human retinal capillary endothelial cells cultured, respectively, in NG and HG were harvested, seeded into 24-well plates (5 × 10^4^ cells/well), and incubated at 37°C for 24 h in the presence of 5% CO_2_. The corresponding medium or different concentrations of BJ (50 and 100 μg/mL) were added for another 24 h of incubation. The HRCRCs were then washed with PBS three times, and 150 μL of 4% paraformaldehyde was added to fix for 30 min at room temperature. After abandoning the supernatant, 150 μL of 0.1% Triton X-100 was added and the samples were incubated for 10 min. Finally, 150 μL of DAPI solution was added to each well and incubated for 10 min at room temperature in the dark. The samples were photographed by the Operetta CLS high-content analysis system.

### Intracellular Reactive Oxygen Species Detection

Intracellular ROS was detected with a ROS assay kit. Briefly, HRCECs cultured, respectively, in NG and HG were harvested, seeded into 6-well plates (5 × 10^5^ cells/well), and incubated at 37°C for 24 h in the presence of 5% CO_2_. DCFH-DA was added and the samples were incubated for 0.5 h. The different concentrations of BJ (50 and 100 μg/mL) or corresponding medium were added for 6 h, and the Beckman flow cytometer was used for detection.

### Western Blot

Human retinal capillary endothelial cells cultured, respectively, in NG and HG were harvested, seeded into six-well plates (5 × 10^5^ cells/well), and incubated at 37°C for 24 h in the presence of 5% CO_2_. The different concentrations of BJ (50 and 100 μg/mL) and the corresponding medium were added for another 24 h of incubation. NP-40 cell lysate was added to split the cells. Protein concentration was determined with a BCA Protein Concentration Assay Kit. The proteins were separated by 10% SDS-polyacrylamide electrophoresis, transferred to PVDF membrane, and sealed with 5% BSA at room temperature for 1 h. After incubation with the corresponding primary antibody overnight at 4°C, the membrane was washed three times with TBST, and the second antibody was used for another 1 h of incubation at room temperature. Then, the membrane was washed three times with TBST. Finally, the protein bands were detected and photographed using the Chemi Scope Mini (Tanon-4600SF).

### Tandem Mass Tag Quantitative Proteomic Analysis

Human retinal capillary endothelial cells cultured, respectively, in NG and HG were seeded into 100 mm culture dishes. When the cells were about 60% confluent, 100 μg/mL BJ was added. After 24 h, NP-40 cell lysate was added to split the cells. Protein concentration was determined with a BCA Protein Concentration Assay Kit. Subsequently, 300 μg of protein was taken from each sample, diluted to 100 μL with PBS, and 500 μL of pre-cooled acetone was added. After mixing, the protein was placed in a −20°C refrigerator and frozen overnight. Then, the supernatant was discarded by centrifugation, 500 μL precooled acetone was added, protein precipitate was collected by centrifugation, and concentrated by vacuum for 5 min. A total of 20 μL of UA solution (8 M urea, 100 mM Tris, PH 7.6) was added and dissolved at room temperature for 1.5 h. A total of 50 mM DTT solution was added until a final concentration of 10 mM, and placed at 30°C for 1.5 h. IAA buffer was added (500 mM IAA in 50 mM TEAB) until a final concentration of 55 mM was obtained, left at room temperature for 40 min avoiding light, and then 50 mM TEAB was added until the urea concentration of lower than 1 M was obtained. Trypsin buffer (4 μg Trypsin in 40 μL and 50 mM TEAB buffer, 1:50 ratio of Trypsin: protein) was added and placed at 37°C for 16–18 h. The C18 Cartridge (3 M, 7 mm/3 ml) was used to desalt the peptide, which was lyophilized and redissolved with 40–50 uL 0.1% formic acid solution, and the thermo quantitative colorimetric assay was performed. This was done by adding 100 μL 50 mM TEAB buffer to the lyophilized sample, which was vortexed mixed, followed by the addition of a 50 μL sample to a 1.5 mL Ep tube for labeling reaction, which was performed by adding and vortex mixing 50 μL 50 mM TEAB, and vortex mixing. After balancing the Tandem mass tag (TMT) reagent to room temperature, 41 μL anhydrous acetonitrile was added, vortex mixed for 5 min, and centrifugated. A total of 41 μL TMT reagent was then added to the sample, mixed vortically, and placed at room temperature for 1 h. The reaction was stopped by the addition of 8 μL 5% hydroxylamine for 15 min, all samples were merged into a 1.5 mL Ep tube, lyophilized after desalting (3 m, 7 mm/3 mL), and stored at −80°C. The samples were separated by reversed-phase chromatography and analyzed by LC-MS/MS. Proteins with fold changes of quantification >1.5 and *P*-value < 0.05 were considered as differential expression. After obtaining the differentially expressed proteins, GO/KEGG analyses were performed to describe the related functions, and the interaction network analysis was also carried out by using the STRING database.

### Statistical Analysis

All data were analyzed using the SPSS 25.0 statistical software, and the data are expressed as mean ± standard deviation. The comparison of the mean between two groups was performed by *T*-test, and the comparison of multiple groups was performed by one-way analysis of variance. *P* < 0.05 was considered statistically significant.

## Results

### Main Components of Bie-Jia-Ruan-Mai-Tang

**FIGURE 1:**
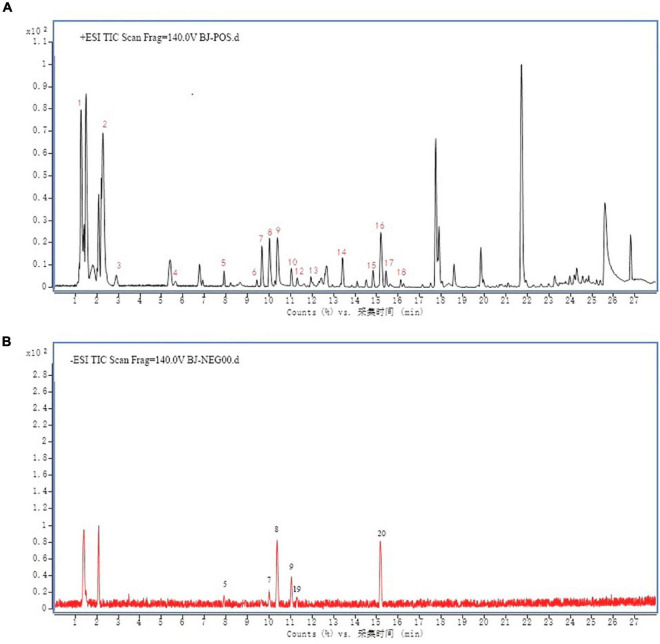
Twenty compounds from BJ were detected by LC-MS/MS analysis in positive and negative ion mode. The chromatographic conditions were as follows: **(A)** Positive ion mode. **(B)** Negative ion mode. Agilent 6530 quadrupole-time of flight mass spectrometry (Q-TOF-MS) system, ACQUITY UPLC HSS T3 (2.1 mm × 150 mm, 1.7 μm) column temperature 60°C. 1. Luteolin, 2. L-tert-Leucine, 3. Vanillic acid, 4. 5-Hydroxymethylfurfural, 5. Loganic acid, 6. Loganin, 7. Sweroside, 8. Albiflorin, 9. Paeoniflorin, 10. Calycosin 7-O-Glucoside, 11. isoliquiritin apioside, 12. Liquiritigenin, 13. Isorhamnetin, 14. Cynaroside, 15. Dipsacoside B, 16. Akebia saponin D, 17. Benzoylpaeoniflorin, 18. Apigenin, 19. Liguiritigenin-7-O-D-apiosyl-4′-O-D-glucoside, 20. Quercitrin.

Bie-Jia-Ruan-Mai-Tang consists of eight raw herbal materials, whose chemical composition is rather complex, so it is necessary to identify its major ingredients. The main components of extract (BJ) were detected by HPLC-Q-TOF-MS in positive and negative ion mode. As demonstrated in Figure 1, a total of 20 compounds were identified as follows: Luteolin (1), L-tert-Leucine (2), Vanillic acid (3), 5-Hydroxymethylfurfural (4), Loganic acid (5), Loganin (6), Sweroside (7), Albiflorin (8), Paeoniflorin (9), Calycosin 7-O-Glucoside (10), isoliquiritin apioside (11), Liquiritigenin (12), Isorhamnetin (13), Cynaroside (14), Dipsacoside B (15), Akebia saponin D (16), Benzoylpaeoniflorin (17), Apigenin (18), Liguiritigenin-7-O-D-apiosyl-4′-O-D-glucoside (19), and Quercitrin (20).

### Bie-Jia-Ruan-Mai-Tang Does Not Reduce Blood Glucose in Diabetic Mice

**FIGURE 2:**
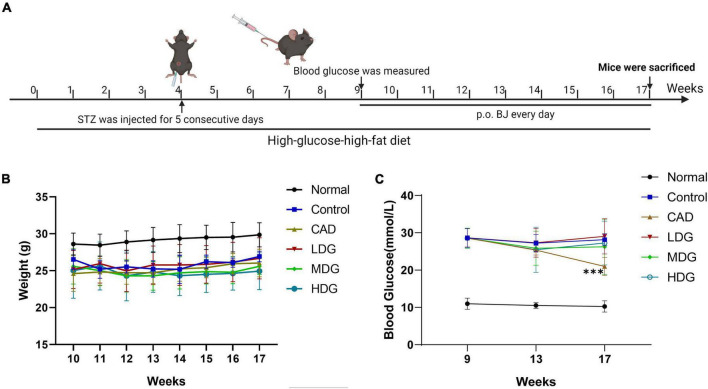
Effect of BJ on the bodyweight and blood glucose in diabetic mice. **(A)** *In vivo* experiment process. Mice were fed a high-fat–high-glucose diet for 17 weeks. In the 4th week, mice were intraperitoneally injected with 50 mg/kg STZ for 5 consecutive days. In the 9th week, mice with blood glucose concentration above 16.7 mmol/L were considered type 2 diabetic. BJ was given for 8 consecutive weeks. Mice were weighed once a week **(B)**, and in the 13th and 17th week, the blood glucose was measured **(C)**. Data are represented as means ± SD (*n* = 8). ***P < 0.001 vs. control.

The *in vivo* experiment process is displayed in Figure 2A. During the experiment, the body weight of mice was measured weekly. As shown in Figure 2B, the weight of mice in the normal group was higher than that of diabetic mice, while there was no obvious difference among the control, CAD, and BJ groups. The bodyweight of each group remained stable during the experiment. As shown in Figure 2C, in the 9th week, the diabetic mice were grouped randomly and hierarchically according to blood glucose concentration, and there was no significant difference in blood glucose between each group. The blood glucose concentration was measured at the 4th week and at the end of the experiment after the administration of the drugs. The results showed that after 8 weeks of administration, the blood glucose of mice in the CAD group decreased markedly, while BJ had no significant effect on blood glucose in diabetic mice.

### Bie-Jia-Ruan-Mai-Tang Inhibits Retinal Angiogenesis in Diabetic Mice

**FIGURE 3:**
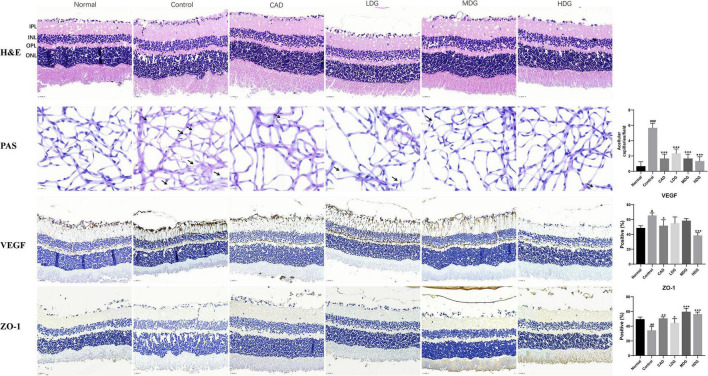
Bie-Jia-Ruan-Mai-Tang (BJ) inhibits retinal angiogenesis in diabetic mice. The H and E, PAS, and immunohistochemistry staining were used to investigate the effects of BJ on retinal structure, acellular capillaries formation, and related protein expression in diabetic mice. The acellular capillaries in each field were counted, and the immunohistochemistry staining for VEGF and ZO-1 was quantified by calculating the positive area. IPL: inner plexiform layer, INL: inner nuclear layer, OPL: outer plexiform layer, ONL: outer nuclear layer. The arrows indicate acellular capillaries. Data are represented as mean ± SD (*n* = 8). ^#^*P* < 0.05, ^##^*P* < 0.01, ^###^*P* < 0.001 vs. Normal; *P < 0.05, **P < 0.01, ***P < 0.001 vs. control.

To investigate the effects of BJ on retinal structure, acellular capillaries formation, and related protein expression in PDR mice, H and E, PAS, and immunohistochemistry techniques were carried out; the results are displayed in Figure 3. Compared with the normal group, the layers of retinal structures in the control group were blurred, and the outer nuclear layer (ONL) and inner nuclear layer (INL) were arranged loosely. However, these changes were reversed after 8 weeks of BJ administration. PAS staining was used to analyze the formation of acellular capillaries in the retina, and the number of acellular capillaries in each field was counted. As a result, the number of acellular capillaries was significantly increased in the control group when compared with the normal group, but sharply reduced by BJ in a dose-dependent manner, suggesting that BJ inhibited the formation of acellular capillaries in the retina. The immunohistochemical staining was quantified by calculating the positive area, and the result exhibited that the expression of VEGF decreased and tight junction protein ZO-1 increased after 8 weeks of BJ administration.

### Bie-Jia-Ruan-Mai-Tang Decreases the Proliferation of High-Glucose-Exposed Human Retinal Capillary Endothelial Cells

**FIGURE 4:**
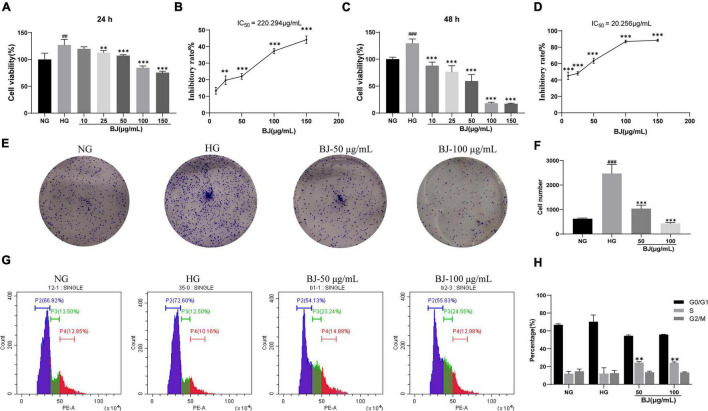
Bie-Jia-Ruan-Mai-Tang (BJ) represses the viability and proliferation of high-glucose-exposed HRCECs. After treatment with BJ for 24 h **(A)** and 48 h **(C)**, respectively, the CCK-8 kit was used to detect the cell viability of HRCECs (*n* = 6). The corresponding IC_50_ values were calculated according to the results of the cell counting assay, which were 220.294 μg/mL **(B)** and 20.256 μg/mL **(D)**, respectively. Cell colony formation assay **(E,F)** was performed to detect the proliferation of cells (*n* = 3). Cell cycle analysis **(G,H)** indicated that high-glucose-exposed HRCECs were arrested at the S phase by BJ (*n* = 3). HRCECs cultured with normal-glucose (NG) were used as control. All data are expressed as mean ± SD. ^##^*P* < 0.01, ^###^*P* < 0.001 vs. NG; **P < 0.01, ***P < 0.001 vs. HG.

The assays for cell counting and plate cloning were used to verify the inhibitory effect of BJ on the viability and proliferation of HRCECs exposed to high glucose. High glucose significantly promoted cell viability, which could be reversed after treatment with different concentrations of BJ for 24 h (Figure 4A) and 48 h (Figure 4C). The IC_50_ values of BJ for 24 h and 48 h were calculated according to the cell-counting assay, which were 220.294 and 20.256 μg/mL, respectively (Figures 4B,D). In the plate cloning test, after treatment with BJ for 8 consecutive days, HRCECs were stained with crystal violet for 15 min and the purple area was counted. As revealed in Figures 4E,F, BJ markedly inhibited the proliferation of HRCECs in a dose-dependent manner. Cell cycle arrest plays an important role in the inhibition of cell proliferation. Therefore, we performed cell cycle analysis to further evaluate the effect of BJ in high-glucose-cultured HRCECs. Propidium iodide (PI) is a fluorescent dye that can produce fluorescence after binding with double-stranded DNA, and the fluorescence intensity is proportional to the content of double-stranded DNA. After the intracellular DNA is stained with PI, the DNA content of the cells is determined by flow cytometry, and then the cell cycle can be analyzed according to the DNA content. As expected, the cell cycle was arrested in the S phase after treatment with different concentrations of BJ for 24 h (Figures 4G,H), indicating that BJ inhibited the proliferation of HRCECs exposed to high glucose through arresting the cell cycle.

### Bie-Jia-Ruan-Mai-Tang Promotes Apoptosis of High-Glucose-Exposed Human Retinal Capillary Endothelial Cells

**FIGURE 5:**
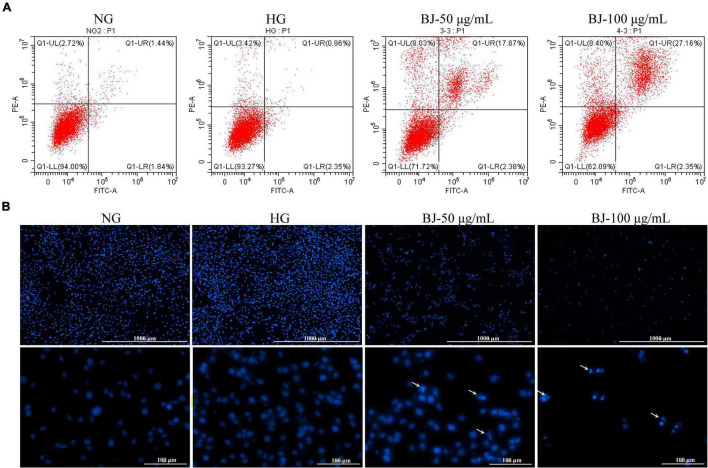
Bie-Jia-Ruan-Mai-Tang (BJ) induces apoptosis of HRCECs exposed to high glucose. **(A)** The apoptosis rate was measured by the flow cytometry after AnnexinV-FITC/PI staining. **(B)** Nuclear morphology was detected by cell nuclear staining, and BJ enhanced the nuclear condensation and fragmentation. The arrows indicate nuclear condensation and fragmentation.

Apoptosis can also reduce the number of cells; therefore, a flow cytometry analysis was used to detect the apoptosis of HRCECs cultured with high glucose after treatment with different concentrations of BJ for 24 h. Annexin V labeled with FITC fluorescent probe was employed to detect apoptosis, and the FITC positive cells were considered apoptotic cells. The results indicated that BJ increased apoptosis of the cells in a dose-dependent manner compared with the HG group (Figure 5A). We further evaluated the effect of BJ on nucleus morphology by DAPI staining, which can produce high-intensity fluorescence by binding to DNA. Therefore, the morphology of the nucleus can be observed through a fluorescence microscope. The nucleus is oval under normal circumstances, but showed irregular shapes after BJ treatment for 24 h (Figure 5B), indicating that BJ promoted the nuclear condensation and fragmentation.

### Bie-Jia-Ruan-Mai-Tang Increases Mitochondrial Dysfunction of High-Glucose-Exposed Human Retinal Capillary Endothelial Cells

**FIGURE 6:**
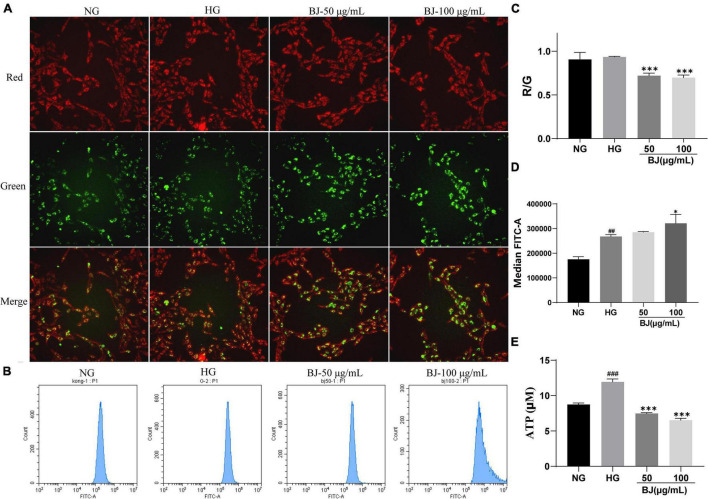
Bie-Jia-Ruan-Mai-Tang (BJ) elicits mitochondrial dysfunction. **(A)** Representative images. JC-1 staining was used to evaluate the MMP change after treatment of the cells with BJ. The normal MMP shows red fluorescence, while green fluorescence represents the decreased membrane potential, indicating mitochondrial dysfunction. The ratio of red to green fluorescence was quantified **(C)**. The level of ROS in HRCECs was measured by a flow cytometer **(B,D)**. The ATP concentration was detected by the ATP assay kit **(E)**. Data are represented as mean ± SD (*n* = 3). ^##^*P* < 0.01, ^###^*P* < 0.001 vs. NG; *P < 0.05, ***P < 0.001 vs. HG.

Mitochondrial membrane potential (MMP) is an indicator of mitochondrial membrane permeability, which is decreased during early apoptosis (16). MMP changes can be detected with the fluorescent probe JC-1 and are high under normal circumstances. After BJ treatment, the Operetta CLS high-content analysis system was used to observe the red and green fluorescence in the HRCECs, and the ratio of red to green fluorescence was quantified. After 24 h of BJ treatment, the proportion of green fluorescence evidently increased, suggesting the reduction of MMP in HRCECs (Figures 6A,C). ATP production is closely related to mitochondrial function (17). Besides, the production of ROS is closely associated with mitochondria as well (18), which are composed of superoxide radical anions, hydrogen peroxide (H_2_O_2_), and hydroxyl radicals. Excessive ROS causes DNA damage and cell death (19). Therefore, we further evaluated the effects of BJ on mitochondria by detecting the production of ATP and ROS in HRCECs. It was found that high glucose markedly promoted the production of ROS and ATP. After treatment with BJ, ROS increased obviously (Figures 6B,D), while ATP (Figure 6E) significantly decreased when compared with the HG group.

### Bie-Jia-Ruan-Mai-Tang Depresses PI3K/AKT Signal Pathway Activation

**FIGURE 7:**
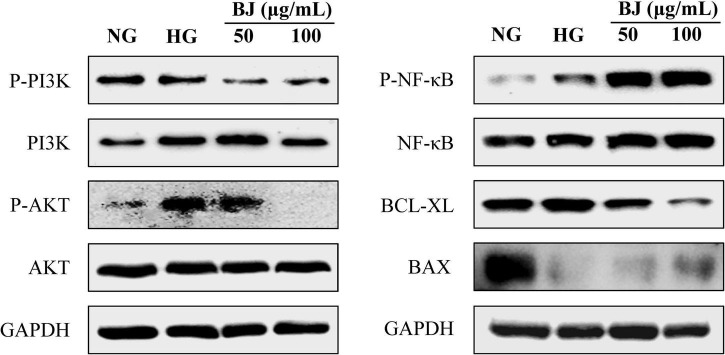
Effect of BJ on protein expression of PI3K/AKT and NF-κB signal pathways in HRCECs exposed to high glucose. Representative images are displayed for p-PI3K, PI3K, p-AKT, AKT, p-NF-κB, NF-κB, Bax, Bcl-xL, and GAPDH protein bands.

Western blot was performed to study the mechanism of BJ against HRCECs exposed to high glucose. As shown in Figure 7, BJ significantly inhibited the expression of p-PI3K and p-AKT when compared with the HG group, indicating that the proliferation inhibition of HRCECs by BJ was related to suppressing activation of the PI3K/AKT signal pathway. Besides, apoptosis is also associated with the NF-κB signaling pathway (20), hence, the expression of NF-κB was detected. The results revealed that BJ significantly promoted the phosphorylation of NF-κB. As BJ was responsible for the collapse of MMP, we examined the expression of mitochondrial apoptosis-related proteins. As a result, high glucose promoted the expression of BCL-XL and inhibited BAX expression, while BJ treatment reversed these changes.

### TMT Quantitative Proteomic Analysis

TMT quantitative proteomic analysis was carried out to detect the differentially expressed proteins after treatment with BJ. The screening conditions were set as *p*-value < 0.05 and quantification fold changes >1.5. After 100 μg/mL BJ treatment for 24 h, 403 differentially expressed proteins were screened out, of which 335 were downregulated and 69 upregulated as compared with the control. Then, the analyses of GO (Gene Ontology), PPI (Protein–Protein Interaction Network Analysis), and KEGG (Kyoto Encyclopedia of Genes and Genomes) were performed to find out the main functions of these differential proteins and the potential targets of BJ.

**FIGURE 8:**
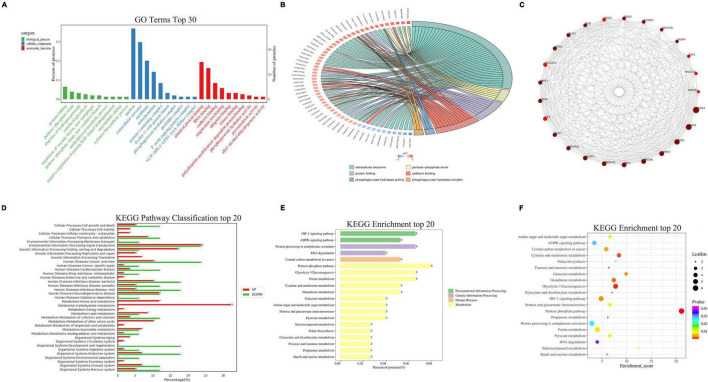
TMT quantitative proteomic analysis. The differentially expressed proteins were divided into three categories by GO analysis, and each category was divided into multiple items. Then top 10 differential proteins in each category were mapped **(A)**. **(B)** GO enrichment chord graph. Red on the left indicates upregulated proteins while blue downregulated proteins. The right represents the GO corresponding items. **(C)** PPI network analysis. Twenty-five proteins with the highest connectivity were selected to draw the interaction network diagram. **(D)** KEGG pathway classification. The percentage of differentially expressed proteins in pathways and metabolism is indicated. **(E,F)** Top 20 pathways and metabolisms with the highest enrichment scores in KEGG enrichment analysis.

Gene Ontology analysis divided the function of protein into three parts, *viz*. cellular component, molecular function, and biological process. The function of most differential proteins is related to cellular component, among which, the main proteins are related to the nucleus, extracellular exosome, and membrane (Figure 8A). The GO enrichment chord diagram shows the GO term involved in the differential proteins (Figure 8B), indicating that their functions are mainly related to extracellular exosomes and cadherin binding. String database can be used to predict functional correlations between proteins. PPI are composed of proteins interacting with each other to participate in biological signal transmission, gene expression regulation, energy and substance metabolism, cell cycle regulation, and other life processes. The differentially expressed proteins were analyzed using the String database to obtain the interaction between them. Twenty-five proteins with the highest connectivity were selected to draw the interaction network diagram (Figure 8C), and the top five proteins in connectivity were listed as follows: HSPA4, ENO1, SOD1, PARK7, and PRDX6. KEGG analysis can help to understand the pathways changed after BJ treatment by analyzing the signaling pathways that are significantly enriched in differentially expressed proteins. KEGG enrichment analysis demonstrated that most of the differential proteins were related to metabolism, including the pentose phosphate pathway, glycolysis, and purine metabolism (Figures 8D–F). As BJ reduced the ATP production, we speculate that BJ inhibits the proliferation of the cells by regulating cell metabolism.

## Discussion

The incidence of DR is continuing to rise (21). There are two main types of DR: early non-proliferative diabetic retinopathy (NPDR) and proliferative diabetic retinopathy (PDR). The main features of NPDR include microaneurysms, retinal hemorrhage, intraretinal microvascular abnormalities (IRMA), and changes in venous diameter, while PDR is characterized by pathological preretinal neovascularization (22). The abnormal proliferation of retinal vascular endothelial cells is closely related to PDR. Since its onset occurs after many years of diabetes progression, there is an opportunity to take steps to prevent vision loss (23). Therefore, drugs that inhibit retinal vascular endothelial cell proliferation are expectedly used to prevent further deterioration of DR. PDR is closely associated with the formation of acellular capillaries, which is obviously promoted by VEGF (24, 25). Additionally, hyperglycemia also impairs the expression of tight-junction protein zonula occludens-1 (ZO-1) in the retina, which will increase the permeability of retinal capillaries, leading to blood extravasation and deterioration of DR (26). In this study, a diabetes model of mice was established through feeding a high-fat–high-glucose diet and intraperitoneal injection of STZ. The results showed that BJ significantly inhibited the formation of acellular capillaries in the retina of model mice, reduced the expression of VEGF, and promoted the expression of ZO-1, thus hindering the development of DR. After we conferred with the relevant literature, calcium dobesilate (CAD) was selected as a positive drug *in vivo* experiments (27, 28), which has antioxidant, free radical, and vascular protection effects. Many randomized controlled clinical trials have confirmed the efficacy and safety of CAD in the treatment of DR (29). In this study, CAD reduced the concentration of blood glucose, thus inhibited the formation of acellular capillaries and the expression of VEGF in the retina of model mice, indicating that CAD has a protective effect on retinal lesions. The *in vitro* experiments were carried out further to explore the related mechanism of action. HRCECs cultured in high-glucose condition were used to mimic DR *in vitro*. High glucose can obviously promote cell proliferation. Bie-Jia-Ruan-Mai-Tang was found to possess the abilities of proliferation inhibition and apoptosis induction in HRCECs exposed to high glucose.

BJ, a Chinese medicine formula composed of eight raw herbal materials, has been verified to have a good therapeutic effect on DR after decades of clinical application. Some of these raw herbal materials have previously been reported to be effective in treating diabetes as well. Turtle shell decoct pill, a formula with the shell of *Trionyx sinensis* as King medicine, can inhibit tumor angiogenesis (30), indicating that the shell possesses the potential to treat vascular diseases. Besides, the formula comprising *Astragalus mongholicus* Bunge and *Panax notoginseng* (*Burkill*) F.H. Chen protects the kidney from inflammatory damage in diabetic nephropathy, possibly by inhibiting mTOR and activating PINK1/Parkin signaling to promote autophagy (31). There are 20 compounds detected in BJ, and many of them have previously been reported to have therapeutic effects on diabetes. Akebia saponin D prevents renal injury in diabetic mice through activating the NRF2/HO-1 pathway and inhibiting the NF-κB pathway (32). Quercitrin can significantly reduce fasting blood glucose concentration and increase insulin level to improve the antioxidant status of diabetic rats (33). Isorhamnetin has a renal protective role by regulating autophagy epigenetic regulators in type 2 diabetes model rats (34). Vanillic acid regulates diabetic hypertension by adjusting blood glucose, insulin, and blood pressure (35). Luteolin alleviates inflammation and oxidative stress through inhibiting NF-κB and upregulating Nrf2, thereby promoting wound recovery in diabetic rats (36). Apigenin ameliorates diabetic nephropathy by depressing oxidative stress and the MAPK pathway (37).

**FIGURE 9:**
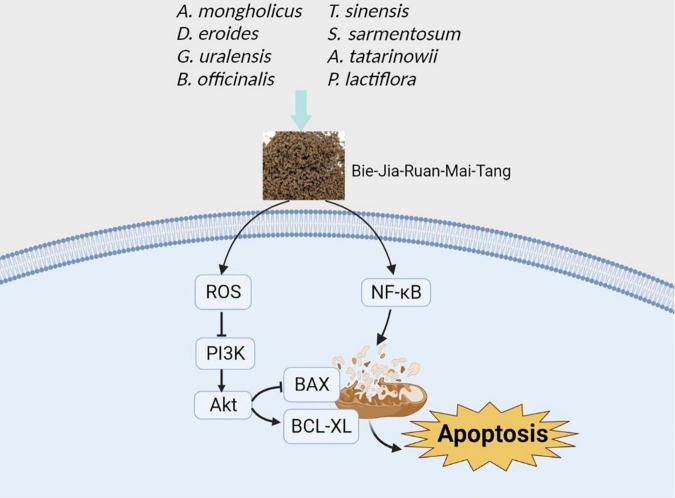
Suggested mechanism of action of BJ eliciting the apoptosis of HRCECs exposed to high glucose. Created with BioRender.com.

PI3K-dependent AKT activation affects several downstream pathways, which involves cell proliferation, angiogenesis, senescence, apoptosis, and cell survival (38, 39). BCL-XL and BAX are associated with the mitochondrial apoptosis pathway. Under normal circumstances, the apoptotic regulator BAX is mediated primarily by continuous reverse-transcriptional translocation elicited by BCL2L1/BCL-XL from mitochondria to the cytoplasm, thus avoiding the accumulation of BAX on the mitochondrial outer membrane (40). BCL-XL is an anti-apoptotic protein belonging to the Bcl-2 family, which helps to maintain the normal membrane state under stress conditions through direct pore-forming of the mitochondrial outer membrane (41). NF-κB, a type of DNA-binding eukaryotic cell transcription factor, is involved in normal physiological processes such as immune and inflammatory responses. The NF-κB signaling pathway is closely related to the development, proliferation, differentiation, and apoptosis of immune cells, and plays a major role in the regulation of inflammatory cytokine gene expression (42). Besides, NF-κB promotes apoptosis by triggering a series of events (24). The present investigation indicates that BJ promotes apoptosis of HRCRCs exposed to high glucose, possibly through inhibition of PI3K/AKT signaling. Furthermore, BJ induces the mitochondrial dysfunction by interfering with the expression of mitochondrial function-related proteins, such as BAX and BCL-XL (Figure 9).

Proteome provides the current expression level of protein molecules, which can help us to understand the mechanism of action of medicines (43). One of the leading causes for diabetes is persistent hyperglycemia. We performed proteomics research on high-glucose-incubated HRCECs after BJ treatment. KEGG enrichment analysis showed that most of the differential proteins were related to metabolism, which was mainly involved in glucose metabolism, such as the pentose phosphate pathway, galactose metabolism, and pyruvate metabolism (Figure 8E). In addition, the pentose phosphate pathway has a higher enrichment score and lower *p*-value (Figure 8F). This suggests that BJ plays a therapeutic role probably by interfering with intracellular glucose metabolism. Although BJ displayed no hypoglycemic effect on the peripheral blood of model mice *in vivo* experiments, this does not prevent its therapeutic effect on PDR.

Currently, there still exist many limitations in the treatment of PDR with surgery and chemicals, such as many adverse reactions and expensive fees. Chinese medicines have attracted great attention owing to their good efficacy, low toxicity, and convenient use. Our study displayed that BJ retarded the development of DR by inhibiting the proliferation of acellular capillaries and promoting the stability of the retina. To further investigate the related mechanism of action, we established an *in vitro* model of retinal endothelial cells cultured with high glucose. The findings indicated that BJ inhibited proliferation and induced apoptosis in the cells by eliciting cycle arrest and decreasing mitochondrial membrane potential *via* inactivation of the PI3K/AKT signaling pathway and activation of the NF-κB pathway.

## Conclusion

Bie-Jia-Ruan-Mai-Tang inhibits the formation of acellular capillary in the retina and the instability of retinal structure in DR mice, suppresses proliferation, and induces apoptosis in HRCECs exposed to high glucose through inducing cell cycle arrest and reducing mitochondrial membrane potential, indicating that BJ has the potential to treat DR.

## Data Availability Statement

The data presented in the study are deposited in the ProteomeXchange repository, accession number PXD012393.

## Ethics Statement

The animal study was reviewed and approved by the Experimental Animal Ethics Committee of Shanghai University of Traditional Chinese Medicine.

## Author Contributions

HZ, XL, and LZ contributed to the conception and design of the experiments. Q-PL, Y-YC, and Y-YY performed the experiments and wrote the draft. PA, Y-ZX, and H-XY performed the statistical analysis and interpreted the data. Y-JZ and LZ provided the raw herbal materials. HZ and KR revised the manuscript. All authors agreed to be accountable for all aspects of the work, ensuring integrity and accuracy.

## Conflict of Interest

The authors declare that the research was conducted in the absence of any commercial or financial relationships that could be construed as a potential conflict of interest.

## Publisher’s Note

All claims expressed in this article are solely those of the authors and do not necessarily represent those of their affiliated organizations, or those of the publisher, the editors and the reviewers. Any product that may be evaluated in this article, or claim that may be made by its manufacturer, is not guaranteed or endorsed by the publisher.

## Abbreviations

NPDR: non-proliferative diabetic retinopathy
PDR: proliferative diabetic retinopathy
BJ: Bie-Jia-Ruan-Mai-Tang
DR: diabetic retinopathy
HRCECs: human retinal capillary endothelial cells
DB: diabetes
MMP: mitochondrial membrane potential
NG: normal glucose group
HG: high-glucose group
IRMA: intraretinal microvascular abnormalities
ATP: adenosine triphosphate
STZ: streptozotocin
ROS: reactive oxygen species
VEGF: Vascular endothelial growth factor
ZO-1: zonula occludens-1
ONL: outer nuclear layer
INL: inner nuclear layer
CAD: Calcium Dobesilate
LDG: low-dose group
MDG: Medium-dose group
HDG: High-dose group
H and E: hematoxylin and eosin
PAS: Periodic Acid-Schiff.
